# Serum Phosphorus, Serum Bicarbonate, and Renal Function in Relation to Liver CYP1A2 Activity

**DOI:** 10.3390/diagnostics13182996

**Published:** 2023-09-19

**Authors:** Joy Ito, Hector Lemus, Tianying Wu

**Affiliations:** 1 Division of Epidemiology and Biostatistics, School of Public Health, San Diego State University, San Diego, CA 92182, USA; jito@sdsu.edu (J.I.); hlemus@sdsu.edu (H.L.); 2 Moores Cancer Center, School of Medicine, University of California, San Diego, CA 92037, USA

**Keywords:** CYP1A2, caffeine metabolism, renal function, serum phosphorus, serum bicarbonate

## Abstract

The liver plays an important role in normal metabolism and physiological functions such as acid-base balance; however, limited epidemiologic studies have investigated how the liver contributes toward acid-base balance using non-invasive biomarkers. We determined associations between serum biomarkers related to acid-base balance and renal function with liver CYP1A2 activity. We used data from 1381 participants of the 2009–2010 National Health and Nutrition Examination Survey (NHANES) with measurements of serum phosphorus, serum bicarbonate, caffeine intake, caffeine metabolites, and estimated glomerular filtration rate (eGFR). Liver CYP1A2 activity was estimated using urine caffeine metabolite indices, which were calculated as the ratio of one of the urine caffeine metabolites (i.e., paraxanthine and 1-methyluric acid) to caffeine intake. We analyzed associations in the whole data set and in different strata of hepatic steatosis index (HSI) based on different cut-points. We found that serum bicarbonate was positively associated with CYP1A2 activity in the whole data set when comparing persons with bicarbonate at Q4 to Q1 (β = 0.18, *p* = 0.10 for paraxanthine; β = 0.20, *p* = 0.02 for 1-methyluric acid). Furthermore, serum phosphorus was positively associated with CYP1A2 activity only in the stratum of 30 ≤ HSI < 36. Lastly, low eGFR was significantly associated with lower CYP1A2 activity measured with paraxanthine in the whole dataset and in all the strata with HSI < 42; when comparing eGFR < 60 to eGFR > 90, β estimates ranged from −0.41 to −1.38, *p*-values ranged from 0.0018 to 0.004. We observed an opposite trend in the highest stratum (HSI ≥ 42). Non-invasive measurements of serum bicarbonate, serum phosphorus, and eGFR have dynamic associations with CYP1A2 activity. These associations depend on the extent of liver damage and the caffeine metabolite used to assess CYP1A2 activity.

## 1. Introduction

Liver health is strongly associated with metabolic health, as research has shown that metabolic disorders such as insulin resistance and metabolic syndrome are strong risk factors for non-alcoholic fatty liver disease (NAFLD) [1,2]. NAFLD, affecting 30% of the general population, is the primary cause of irreversible cirrhosis and hepatocellular carcinoma, and it is the fastest-growing cause of liver mortality [3,4]. However, NAFLD is often underdiagnosed due to the invasive nature of biopsy and the limited availability of imaging diagnosis for the general population [5]. The liver is a highly regenerative organ, making it crucial to understand liver health prior to a diagnosis of NAFLD [6,7]. This would provide a wider window of time for intervention. Unfortunately, there are currently limited non-invasive biomarkers that can indicate liver health before a diagnosis of NAFLD [5].

The liver is a multi-functional organ responsible for a range of processes, including but not limited to bile production, protein synthesis, gluconeogenesis, and the metabolism of drugs, toxins, carbohydrates, fats, and proteins [8]. The liver’s ability to metabolize substances is crucial for its normal function and can influence other liver functions, such as protein synthesis [9]. Therefore, studying liver metabolism may help us understand subclinical progress among asymptomatic populations. CYP enzymes are responsible for liver metabolism, making them essential for investigation. Although more evidence of CYP1A2 activity in humans is emerging, most studies on CYP enzymes have been conducted on animal liver tissues or cells [10]. One of the CYP enzymes, CYP1A2, is known to metabolize caffeine, drugs such as propranolol, and pre-carcinogens such as aflatoxins [11,12]. Recently, the ratio of circulating caffeine metabolites to caffeine intake has been used as a proxy measure for CYP1A2 activity [13]. Using this proxy measure, our group has shown that CYP1A2 activity can vary significantly among individuals without liver diseases and with normal liver enzyme levels, depending on their smoking status (past, never, or current) [14].

Furthermore, acid-base balance is crucial for the processes of blood oxygenation, protein folding, and cellular respiration [15]. The main organs involved in acid-base regulation are the lungs and kidneys [15,16,17]. The liver also plays a role in acid-base balance; patients with liver dysfunction often present with acid-base disorders in hospitalized or ambulatory settings [17,18]. The known mechanisms explaining how the liver regulates acid-base balance in these populations include lactic acid metabolism, albumin synthesis, the production of keto-acids, and ureagenesis [18]. Limited information exists regarding how the liver contributes toward acid-base regulation among relatively healthy populations without acid-base disorders or extensive metabolic damage [16,18,19].

To address a potential research gap, we plan to investigate the association between acid-base balance and CYP1A2 activity using non-invasive clinical tests. Furthermore, we aim to examine whether these associations vary across different stages of liver damage using a non-invasive index, namely hepatic steatosis index (HSI). This study will use the 2009–2010 National Health and Nutrition Examination Survey (NHANES) data to examine serum bicarbonate, phosphorus, and renal function with CYP1A2 activity. By understanding the relationship between acid-base balance and CYP1A2 activity, we may be able to develop better non-invasive biomarkers for liver health and improve our understanding of liver metabolism.

## 2. Materials and Methods

### 2.1. Study Population

We used a sample from the 2009–2010 NHANES, a cross-sectional survey about health and nutritional status of a nationally representative population of non-institutionalized civilians [20]. Of the 10,537 persons surveyed, 2174 had measurements of urine caffeine metabolites and urine creatinine. In the end, we included 1381 participants who were 18 years of age or older, who ingested at least 20 mg of caffeine in the first 24-h dietary recall, and who had measurements of serum phosphorus and serum bicarbonate, as well as urine caffeine metabolites and urine creatine.

### 2.2. Assessment of General Characteristics through Self Reports

Self-reported questionnaires were used to collect demographic information such as age, race/ethnicity, gender, diabetes diagnosis, and alcohol consumption.

Also, physical activity was determined with total metabolic equivalents (METs) per week based on a participant’s self-reported total amount of vigorous and/or moderate exercise per week. First, we calculated the sum of the total minutes of each category of exercise intensity. This sum was multiplied by the proper MET score prescribed by NHANES [21]. MET scores increase with respect to exercise intensity. Physical activity (MET-minutes per week) was calculated using the following equation: (Minutes of vigorous activity of work per day) * (days of vigorous work activity) * (MET score = 8) + (minutes of moderate activity at work per day) * (days of moderate work activity) * (MET score = 4) + (minutes of vigorous recreational activities per day) * (days of vigorous recreational activities) * (MET score = 8) + minutes of moderate recreational activities per day) * (MET score = 4). MET = minutes per week were converted to MET hours per week. For our analyses, we classified physical activities into three groups at 0, 4 to 40, and 40+. 

Smoking habits were self-reported by participants [22]. Lifetime smoking intensity equaled the total number of cigarettes smoked over a participant’s lifetime. Calculations were done separately for former smokers, current smokers, and people who had never smoked since participants received different questions based on their current smoking status. The lifetime smoking intensity of participants who had never smoked equaled 0. Former smokers’ lifetime smoking intensity = (number of cigarettes smoked per day) * (duration of smoking before quitting (years)) * (365.25 days/year). Current smokers’ lifetime smoking intensity = (total days smoked cigarettes/month) * (average cigarettes/day) * (12 months/year).

### 2.3. General Body Measurements

Body measurements were conducted in the NHANES mobile examination center (MEC); measurements used in our analyses included but were not limited to body mass index (BMI) and blood pressure. Additional details of the process have been documented elsewhere [23,24]. For our analysis, the systolic and diastolic blood pressure measurements were an average of all available measurements. We divided systolic blood pressure by diastolic blood pressure and categorized these measurements into 5 classes: normal, elevated, hypertension stage 1, and hypertension stage 2 according to clinically accepted guidelines in the U.S. [25].

### 2.4. Caffeine Intake Assessment

Caffeine intake was assessed using the first of two 24-h recalls that used a multi-pass method algorithm from the US Department of Agriculture. Participants reported their food and beverage intake in the past 24-h to trained interviewers. The first 24-h recall was conducted in the NHANES MEC. The amount of ingested caffeine (mg) was estimated from consumption of all food and beverages that contained caffeine [26].

### 2.5. Assessment of Urinary Caffeine Metabolite and CYP1A2 Activity

**Figure 1:**
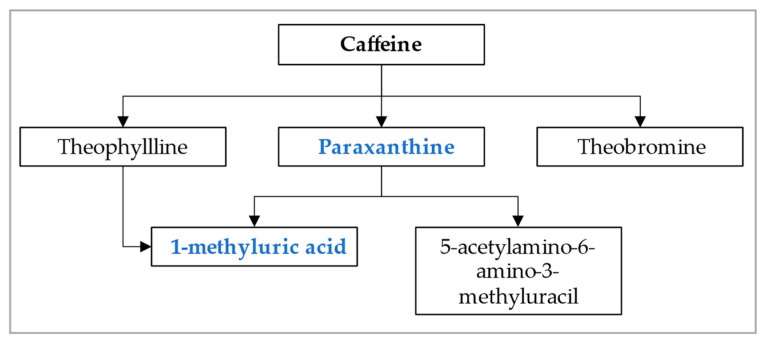
Main caffeine metabolites in humans (adapted from Nehlig et al.) [27].

In the NHANES study, 14 caffeine metabolites were measured; only paraxanthine and 1-methyluric acid were included in our analysis. Paraxanthine is a main upstream metabolite, and 1-methyluric acid is a main downstream metabolite associated with caffeine clearance, as shown in Figure 1 [27]. These metabolites were measured by performing high performance chromatography-electrospray ionization-tandem quadrupole mass spectrometry (HPLC-ESI-MS/MS). Creatinine was measured using the Roche/Hitachi Modular P Chemistry Analyzer. Detailed methods can be found in the NHANES protocol [28]. To determine the urinary concentration of caffeine metabolites (μmol/L), we divided caffeine metabolites by urine creatinine levels. We expressed measurements in μmol/mg). To assess CYP1A2 activity, we created a caffeine metabolite index by using one of the caffeine metabolites to divide the caffeine intakes from the last 24 h on the day of blood draw, which was the first 24-h recall in the NHANES. The caffeine metabolite index is calculated as: (urine creatinine-adjusted caffeine metabolites)/(caffeine intakes from the first 24-h recall).

### 2.6. Serum Bicarbonate and Serum Phosphate Assessment

Serum bicarbonate and serum phosphate concentrations were measured using a DxC800. For serum bicarbonate, an indirect ion sensitive electrode measured the pH rate of change as CO_2_ ions diffused across a membrane. The pH rate of change was proportional to the sample’s CO_2_ levels, producing measurements of CO_2_ in serum. For phosphorus, the DxC800 used a timed-rate method to determine serum phosphorus concentrations. In an acidic solution, inorganic phosphorus reacted with ammonium molybdate to form a colored phosphomolybdate complex. The absorbance at 365 nm was recorded since the absorbance was proportional to the phosphate concentration [29].

### 2.7. Estimated Glomerular Filtration Rate (eGFR)

Estimated glomerular filtration rate (eGFR) was estimated using the Chronic Kidney Disease Epidemiology Collaboration (CKD-Epi equation model using serum creatinine, sex, age, and race). Information regarding this equation can be found elsewhere [30]. We categorized eGFR into the following groups: >90, 60 to 90, and <60 mL/min/1.73 m^2^.

### 2.8. Hepatic Steatosis Index

In 2009, Lee et al. developed the Hepatic Steatosis Index (HSI) as a non-invasive screening measure to approximate NAFLD status in a representative Korean population [31]. HSI was used to detect NAFLD among participants whose HSI score was greater than 36 with 93% sensitivity. NAFLD was ruled out with 92% specificity among participants whose HSI score was less than 30. The AUROC was 0.812. This is the calculation for HSI: 8*(ALT/AST) + BMI (+2 if diabetic) (+2 if female). We calculated HSI scores for each participant and performed stratified analysis by grouping them as follows: HSI < 30, 30 ≤ HSI < 36, 36 ≤ HSI < 42, and HSI ≥ 42. Our cut-offs differed from the original guidelines for Koreans due to the distribution of HSI scores among the NHANES study population, which was expected since BMI and waist circumferences are significantly higher among Caucasians in the U.S. versus Koreans. 

### 2.9. Statistical Analyses

Linear regression was used to assess associations between serum phosphate and serum bicarbonate as well as eGFR with liver CYP1A2 activity, measured by caffeine metabolite indices. As caffeine metabolite indices were not normally distributed, they were log-transformed. Covariate selection was determined on a priori assumption based on general knowledge, practice, and literature in this field. Our covariates were as follows: age, gender, race/ethnicity, blood pressure status, physical activity, blood glucose levels, lifetime smoking intensity, BMI, and alcohol intake. Stratified analyses by HSI status were also performed to determine above associations. We evaluated these associations using multivariable linear regression model with adjustment of weight and clusters used in survey research. We used SAS 9.4 to perform all statistical analyses.

## 3. Results

### 3.1. Demographic Characteristics in the Whole Data Set (Table 1) and by HSI Status (Table 2)

In the whole data set (Table 1), the study population was comprised of roughly equal numbers of males and females, with approximately 30–36% of participants falling into each of the following age groups: 18–39, 40–59, ≥60. Half of the participants were white and had never smoked, and more than 70% were either overweight or obese. At least half of the participants had elevated blood pressure, 10% had diabetes, and more than 35% had an eGFR of 60 or less. Nearly 30% of participants were sedentary, while 30% reported a MET-hour/week of 40 or greater.

**Table 1:** Characteristics of participants enrolled in this study in the whole dataset.

Characteristic	Total Cohort (*n* = 1381)
Gender	
Female	707 (51%)
Male	674 (49%)
Age	
18 to 39	458 (33%)
40 to 59	493 (36%)
60 or older	430 (31%)
Ethnicity	
Mexican American	246 (18%)
Other Hispanic	143 (10%)
White Non-Hispanic	732 (53%)
Black Non-Hispanic	205 (15%)
Other	55 (4%)
Smoking intensity, total cigarettes over lifetime	
0	688 (53%)
1 to 50,400	252 (19%)
>50,400	364 (28%)
BMI, kg/m²	
<25	394 (29%)
25 to 29.9	460 (33%)
>29.9	527 (38%)
Blood pressure status	
Normotensive	659 (48%)
Elevated	255 (18%)
Hypertension 1	246 (18%)
Hypertension 2	221 (16%)
Diabetes	
Yes	133 (10%)
No	1212 (88%)
Borderline	33 (2%)
eGFR, mL/min/1.73 cm²	
>90	902 (65%)
60 to 90	395 (29%)
<60	84 (6%)
Physical activity, MET-hours/week	
0	373 (27%)
>0 to 4	93 (7%)
>4 to 40	496 (36%)
>40	419 (30%)
Paraxanthine/caffeine ratio	0.15 (0.07, 0.30)
1-methyluric acid/caffeine ratio	0.59 (0.33, 1.07)
Bicarbonate level, mmol/L	26 (24, 27)
Phosphorus level, mmol/L	26 (24, 27)

Categorical variables are presented as *n*, % (column %), based on known values; continuous variables are presented as median and interquartile range. Caffeine metabolite indices were multiplied by 1000. List of abbreviations: SD: standard deviation, BMI: body mass index, eGFR: estimated glomerular filtration rate, MET: metabolic equivalent.

In addition, we examined the distribution trends of these characteristics across HSI strata (Table 2). We found that as the HSI strata increased, the percentage of individuals in the 40–59 age group increased; there was an increase in the prevalence of obesity, diabetes, and stage 1 and stage 2 hypertension among Mexican Americans. Individuals with an eGFR > 90 were more prevalent in higher HSI strata; meanwhile, they had slightly reduced levels of bicarbonate, phosphorus, and levels of the caffeine index using 1-methyluric acid.

**Table 2:** Characteristics of participants enrolled in this study stratified by hepatic steatosis index (HSI) status.

Characteristic	HSI < 30 (*n* = 197)	30 ≤ HSI < 36 (*n* = 393)	36 ≤ HSI < 42 (*n* = 432)	HSI ≥ 42 (*n* = 359)
Gender				
Female	104 (53%)	179 (46%)	215 (50%)	209 (58%)
Male	93 (47%)	214 (54%)	217 (50%)	150 (42%)
Age				
18 to 39	88 (45%)	141 (36%)	116 (27%)	113 (31%)
40 to 59	54 (27%)	127 (32%)	154 (36%)	158 (44%)
60 or older	55 (28%)	125 (32%)	162 (37%)	88 (25%)
Ethnicity				
Mexican American	16 (8%)	64 (16%)	87 (20%)	79 (22%)
Other Hispanic	20 (10%)	40 (10%)	45 (10%)	38 (11%)
White Non-Hispanic	113 (57%)	220 (56%)	228 (53%)	171 (48%)
Black Non-Hispanic	29 (15%)	50 (13%)	63 (15%)	63 (18%)
Other	19 (10%)	19 (5%)	9 (2%)	8 (2%)
Smoking intensity, total cigarettes over lifetime				
0	89 (50%)	189 (51%)	220 (54%)	190 (55%)
1 to 50,400	40 (23%)	79 (22%)	72 (17%)	61 (17%)
>50,400	48 (27%)	101 (27%)	119 (29%)	96 (28%)
BMI, kg/m²				
<25	195 (98%)	187 (47%)	12 (3%)	0 (0%)
25 to 29.9	1 (1%)	199 (51%)	252 (58%)	8 (2%)
>29.9	1 (1%)	7 (2%)	168 (39%)	351 (98%)
Blood pressure status				
Normotensive	124 (63%)	204 (52%)	186 (43%)	145 (40%)
Elevated	18 (9%)	84 (21%)	88 (20%)	65 (18%)
Hypertension 1	30 (15%)	58 (15%)	69 (16%)	89 (25%)
Hypertension 2	25 (13%)	47 (12%)	89 (21%)	60 (17%)
Diabetes				
Yes	3 (2%)	18 (5%)	54 (12%)	58 (16%)
No	191 (98%)	371 (94%)	370 (86%)	280 (78%)
Borderline	0 (0%)	4 (1%)	8 (2%)	21 (6%)
eGFR, mL/min/1.73 cm²				
>90	125 (63%)	252 (64%)	274 (63%)	251 (70%)
60 to 90	64 (32%)	115 (29%)	120 (28%)	96 (27%)
<60	8 (4%)	26 (7%)	38 (9%)	12 (3%)
Physical activity, MET-hours/week				
0	39 (20%)	83 (21%)	128 (30%)	123 (34%)
>0 to 4	16 (8%)	25 (6%)	28 (6%)	24 (7%)
>4 to 40	77 (39%)	155 (39%)	150 (35%)	114 (32%)
>40	65 (33%)	130 (33%)	126 (29%)	98 (27%)
Paraxanthine/caffeine ratio	0.16 (0.06, 0.32)	0.15 (0.07, 0.31)	0.15 (0.07, 0.30)	0.15 (0.07, 0.28)
1-Methyluric acid/caffeine ratio	0.66 (0.40, 1.14)	0.58 (0.33, 1.07)	0.57 (0.32, 1.06)	0.55 (0.32, 0.99)
Bicarbonate level, mmol/L	26 (25, 27)	26 (25, 27)	25 (24, 27)	25 (24, 27)
Phosphorus level, mmol/L	1.20 (1.10, 1.32)	1.23 (1.10, 1.32)	1.20 (1.07, 1.32)	1.16 (1.07, 1.29)

Categorical variables are presented as *n*, % (column %), based on known values; continuous variables are presented as median and interquartile range. Caffeine metabolite indices were multiplied by 1000. List of abbreviations: SD: standard deviation, BMI: body mass index, eGFR: estimated glomerular filtration rate, MET: metabolic equivalent.

### 3.2. Multivariable Adjusted Associations of Serum Bicarbonate, Serum Phosphorus, and eGFR with CYP1A2 Activity Measured by Caffeine Metabolite Indices (Table 3 and Figure 2 and Figure 3)

Serum phosphorus: Serum phosphorus was not associated with CYP1A2 activity in the whole data set or in most strata, except in strata of 30 ≤ HSI < 36. In the stratum of 30 ≤ HSI < 36, we observed positive and marginally/statistically significant associations for two caffeine metabolites. Comparing quartile 4 to quartile 1 of serum phosphorus, the beta estimate was 0.29 (*p* = 0.10) for paraxanthine and 0.25 (*p* = 0.04) for 1-methyluric acid. 

We further examined the association between serum phosphorus and CYP1A2 activity without the adjustment of eGFR; the associations did not change significantly.

**Table 3:** The associations of caffeine metabolite indices with serum phosphorus and with serum bicarbonate and with estimated glomerular filtration rate (eGFR), adjusting for glucose, gender, ethnicity, age, BMI, blood pressure, physical activity categories, lifetime smoking intensity, and excess of alcohol intake *.

	Overall (*n* = 1381)		HSI < 30 (*n* = 197)		30 ≤ HSI < 36 (*n* = 393)		36 ≤ HSI < 42 (*n* = 432)		HSI ≥ 42 (*n* = 359)	
	Paraxanthine	1-MUA	Paraxanthine	1-MUA	Paraxanthine	1-MUA	Paraxanthine	1-MUA	Paraxanthine	1-MUA
	β (*p*-Value)	β (*p*-Value)	β (*p*-Value)	β (*p*-Value)	β (*p*-Value)	β (*p*-Value)	β (*p*-Value)	β (*p*-Value)	β (*p*-Value)	β (*p*-Value)
Phosphorus, mmol/L										
Quartile 1 (0.55–1.10)	Ref	Ref	Ref	Ref	Ref	Ref	Ref	Ref	Ref	Ref
Quartile 2 (1.13–1.20)	−0.01 (0.94)	−0.02 (0.69)	0.07 (0.80)	−0.09 (0.65)	0.01 (0.96)	−0.02 (0.90)	−0.19 (0.26)	−0.15 (0.25)	0.13 (0.58)	0.11 (0.47)
Quartile 3 (1.22–1.36)	−0.04 (0.58)	0.03 (0.61)	−0.19 (0.51)	0.07 (0.69)	0.04 (0.75)	−0.01 (0.94)	−0.03 (0.87)	0.08 (0.46)	−0.11 (0.48)	0.07 (0.54)
Quartile 4 (1.38–2.62)	0.13 (0.15)	0.11 (0.22)	0.05 (0.90)	0.01 (0.96)	**0.29 (0.10)**	**0.25 (0.04)**	0.05 (0.82)	0.11 (0.51)	0.14 (0.43)	0.03 (0.87)
Bicarbonate, mmol/L										
Quartile 1 (15–24)	Ref	Ref	Ref	Ref	Ref	Ref	Ref	Ref	Ref	Ref
Quartile 2 (25–25)	0.15 (0.21)	0.01 (0.88)	0.29 (0.24)	0.06 (0.72)	0.03 (0.92)	−0.05 (0.74)	0.24 (0.20)	0.20 (0.14)	0.13 (0.58)	−0.12 (0.43)
Quartile 3 (26–26)	0.12 (0.24)	**0.12 (0.10)**	0.33 (0.25)	0.12 (0.65)	0.16 (0.31)	0.15 (0.20)	−0.01 (0.96)	0.08 (0.59)	0.19 (0.36)	0.17 (0.24)
Quartile 4 (27–38)	**0.16 (0.06)**	0.13 (0.12)	0.47 (0.19)	0.12 (0.67)	0.19 (0.28)	0.23 (0.19)	0.04 (0.82)	0.14 (0.33)	**0.37 (0.09)**	0.15 (0.34)
eGFR										
>90	Ref	Ref	Ref	Ref	Ref	Ref	Ref	Ref	Ref	Ref
60 to 90	**−0.23 (0.0031)**	**−0.11 (0.08)**	**−0.64 (0.06)**	**−0.52 (0.03)**	**−0.36 (0.10)**	−0.16 (0.24)	**−0.34 (0.04)**	−0.19 (0.28)	0.14 (0.32)	0.04 (0.78)
<60	**−0.39 (0.0010)**	**0.25 (0.02)**	**−1.38 (0.04)**	−0.62 (0.21)	**−0.61 (0.03)**	0.15 (0.49)	**−0.79 (0.0018)**	0.27 (0.14)	0.68 (0.11)	0.52 (0.23)

* Caffeine metabolites indices were the ratios of caffeine metabolites to caffeine intake, including creatine adjusted paraxanthine/caffeine intake and creatinine adjusted 1-methyluric acid/caffeine intake; indices were log transformed. List of abbreviations: 1-MUA: 1-methyluric acid. Bolded p for trend values indicate significance using a significance level of 0.05.

**Figure 2:**
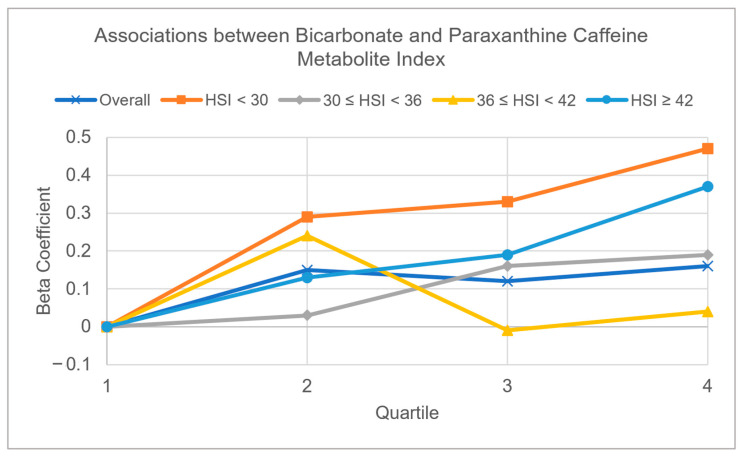
Patterns of serum bicarbonate with paraxanthine caffeine metabolite index in the whole dataset, and four hepatic steatosis index (HSI) strata (HSI < 30, 30 ≤ HSI < 36, 36 ≤ HSI < 42, and HSI ≥ 42). The covariates adjusted in multivariable models included glucose, gender, ethnicity, age, BMI, blood pressure, physical activity, lifetime smoking intensity, and excessive alcohol intake.

Serum bicarbonate: Using paraxanthine to evaluate CYP1A2 activity, the trend of association was mostly positive and marginally significant in the whole data set; the beta estimate was 0.16 (*p* = 0.06) for paraxanthine when comparing quartile 4 to quartile 1. The beta estimate was 0.12 (*p* = 0.10) for 1-methyluric acid when comparing quartile 3 to quartile 1 of bicarbonate. The positive trend was present in all strata but only reached marginal significance in HSI ≥ 42, with a beta estimate of 0.37 (*p* = 0.09) for paraxanthine comparing quartile 4 to quartile 1 (Figure 2). Using 1-methyluric acid to evaluate CYP1A2 activity, the trend was positive for every stratum but was statistically significant only in the whole data set when comparing quartile 4 against quartile 1 (β = 0.20, *p* = 0.02) and was marginally significant when comparing quartile 3 against quartile 1 (β = 0.14, *p* = 0.11).

We further examined the association between serum bicarbonate and CYP1A2 activity without the adjustment of eGFR; the associations did not change significantly.

**Figure 3:**
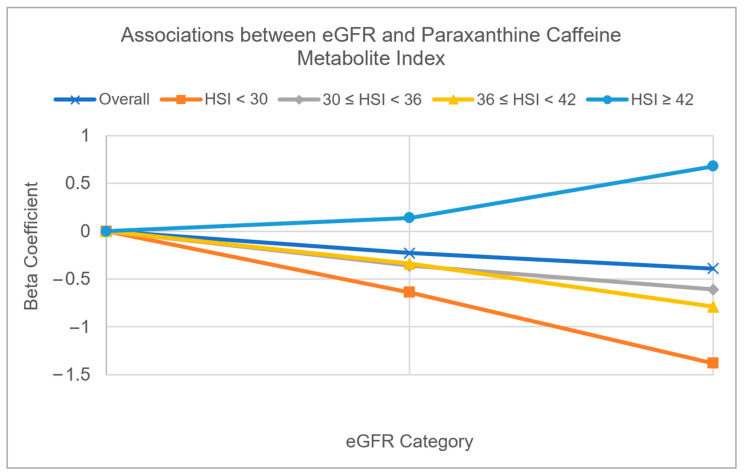
Patterns of estimated glomerular filtration rate (eGFR) with paraxanthine caffeine metabolite index in the whole dataset, and four hepatic steatosis index (HSI) strata (HSI < 30, 30 ≤ HSI < 36, 36 ≤ HSI < 42, and HSI ≥ 42). The covariates adjusted in multivariable models included glucose, gender, ethnicity, age, BMI, blood pressure, physical activity, lifetime smoking intensity, and excessive alcohol intake.

eGFR: Using paraxanthine to evaluate CYP1A2 activity, lower eGFR was associated with reduced CYP1A2 activity in the whole data set. When comparing eGFR <60 to eGFR >90, we obtained a beta estimate of −0.39 (*p* = 0.0010). This trend of association was consistent across most strata of HSI, except the strata of HSI ≥ 42. In the HSI ≥ 42 strata, we found that lower GFR was marginally associated with higher CYP1A2 activity with an estimate of 0.68 (*p* = 0.11) when comparing GFR <60 to GFR >90. Using 1-methyluric acid to evaluate CYP1A2 activity, we did not observe a linear trend of associations between GFR and CYP1A2 activity. However, we did observe that people with a GFR of 60 to 90 had lower CYP1A2 activity as compared to a GFR of >90 (β = −0.11, *p* = 0.08). This type of trend was also observed in strata with HSI < 30 with a beta estimate of −0.52 (*p* = 0.03) when comparing GFR of 60 to 90 to GFR > 90 (Figure 3).

We also examined the same associations in multivariable models without adjusting for phosphorus and bicarbonate. The results did not change considerably.

## 4. Discussion

Using a nationally representative sample in our study, we assessed the associations of serum bicarbonate, serum phosphorus, and eGFR with CYP1A2 activity. We also assessed these associations with different levels of HSI, a non-invasive index to assess liver damage. First, trends between bicarbonate and CYP1A2 activity were positive and significant or marginally significant using either paraxanthine or 1-methyluric acid. The magnitude of this positive trend was strongest in HSI ≥ 42 when using paraxanthine. Second, phosphorus and CYP1A2 activity was only positive and significant in 30 ≤ HSI < 36. Last, lower eGFR was associated with lower CYP1A2 activity in the whole data set and most strata except in stratum with HSI ≥ 42. Conversely, we observed that lower eGFR was associated with a higher CYP1A2 activity in HSI ≥ 42.

We found significant positive associations between bicarbonate and CYP1A2 activity in both caffeine metabolites within the entire dataset. Bicarbonate serves as a base and regulates pH in metabolic processes not only in the kidney but also in the liver. In the liver, bicarbonate can neutralize protons generated during gluconeogenesis and fat and carbohydrate oxidation [18,32]. The liver is the primary organ responsible for producing bile acids, while bicarbonate also contributes to bile production, which aids in fat absorption and digestion [33,34]. All of the above potentially explain the observed positive association between bicarbonate and CYP1A2 activity.

Upon examining the associations between bicarbonate and CYP1A2 activity across different strata, the magnitudes of positive associations varied. In most strata with HSI < 42, the associations were not statistically significant, but there was marginal significance in the HSI ≥ 42 stratum. It is possible that at higher degrees of liver damage (HSI ≥ 42), a greater amount of bicarbonate is needed to aid in liver metabolism. However, a larger study with a more significant sample size is necessary to determine if this relationship is dynamic across different HSI strata or if it is due to chance. We did not find significant changes with or without GFR adjustment, indicating that the kidneys are not a main mediator between bicarbonate and CYP1A2 activity. Although bicarbonate is filtered by the glomeruli and then reabsorbed in the proximal tubes in kidney, the pancreas is primarily responsible for the producing of sodium bicarbonate [35,36].

We did not observe a significant association between serum phosphorus and CYP1A2 activity in the whole data set and most strata of HSI; however, we observed positive and significant/marginally significant associations between phosphorus and CYP1A2 activity in the stratum with 30 ≤ HSI < 36, where participants in this stratum were unlikely to have severe liver damage. Phosphorus is an essential nutrient for the phosphorylation of glucose when glucose enters hepatocytes during glycogen synthesis and is necessary for NAD+ synthesis [37]. Thus, higher levels of phosphorus may increase liver metabolism. Though high levels of phosphorus may increase burden to the kidneys, we found that the association between phosphorus and CYP1A2 activity did not change with or without adjusting for eGFR. This indicates that the association is not affected by renal function.

In this study, we evaluated the associations between kidney function and liver metabolism function in a population, where most were without severe liver or chronic kidney disease. We observed that lower kidney function (lower eGFR) was significantly associated with lower CYP1A2 metabolism, measured by the main and an upstream caffeine metabolite, paraxanthine. This association is consistent in most strata where HSI < 42. Although the liver and kidneys have distinct functions, they work together closely to maintain the body’s overall health. The liver produces urea, a waste product that is eliminated by the kidneys [18,38]. The kidneys also help regulate liver function by maintaining a proper balance of fluids, electrolytes, and bicarbonate [39].

Furthermore, it remains unclear why lower eGFR was marginally associated with higher CYP1A2 activity in the highest stratum of HSI ≥ 42. Our data indicate that higher CYP1A2 activity may not always be linearly related to better liver health but may sometimes be associated with greater liver and kidney damage. Higher CYP1A2 activity could be a self-defense mechanism when there are more toxins due to reduced liver function and more waste that cannot be excreted by the kidneys. The liver has unique regenerative capacities and compensates more when liver damage is more extensive [40]. This compensatory mechanism may lead to higher CYP1A2 activity in individuals with lower GFR at higher HSI stratum. The interaction between liver health and kidney disease warrants further investigation. NAFLD, one of the most common liver diseases, is a strong risk factor for chronic kidney disease (CKD) [41,42]. Liver-related portal inflammation [43] and portal hypertension can trigger a subclinical hepatorenal reflex, which over time may contribute to the development and progression of CKD [44]. Further mechanistic studies are needed in this area to better understand the interrelationship between liver regeneration, eGFR, CYP1A2 activity, and liver damage. These studies could involve examining the role of liver enzymes, other CYP protein activities, and novel kidney damage-related biomarkers.

Several single nucleotide polymorphisms (SNPs) have been associated with the CYP1A2 gene. For instance, the rs2472297 allele has been linked to increased levels of coffee consumption, a reduction in blood pressure, and an increase in eGFR [45]. Increased eGFR typically indicates better renal function. Since the kidney is one of the key organs responsible for regulating acid-base balance [19], we infer that these types of alleles are likely to help regulate acid-base balance.

It is worth noting that there are large variations of SNPs associated with a certain gene, with some studies identifying the rs2472297 allele and others finding another allele (rs3860) associated with the CYP1A2 gene [46,47]. Due to the extensive variation in SNPs, they may or may not reliably determine miRNA and protein expression levels, let alone activity levels; these SNPs require large-scale validations [48]. Some studies have demonstrated that miRNA expression aligns more closely with CYP1A2 activity than SNPs [47]. Consequently, even when using a proxy measure of CYP1A2, we believe this feasible approach will offer more accurate predictions and broader applications than the SNPs of CYP1A2, making it a valuable tool for assessing its relation to acid-base balance.

The study has several strengths, including the use of nationally representative data from NHANES. In addition, the study utilized a non-invasive measure of CYP1A2 activity to evaluate its correlation with clinical biomarkers related to acid-base balance and renal function. Conventional liver enzyme tests cannot reflect liver metabolism during the early stages of liver damage. Moreover, the study investigated the associations between these factors at different stages of liver damage, as measured by HSI, which further enhances the study’s robustness.

The cross-sectional design of the study limits the interpretation of associations as being causal, and reverse causation is also possible. Therefore, causation should be evaluated in longitudinal studies. In addition, there are other pathways that may influence CYP1A2 activity apart from the proposed biomarkers. CYP1A2 activity may correlate with some but not all CYP enzyme activity [49]. Moreover, CYP1A2 was measured using a proxy versus direct measurement. The denominator used for calculating the CYP1A2 proxy relied on dietary caffeine intakes estimated from self-reported foods and beverages, which could have been affected by recall bias or measurement error. It is worth noting that the mechanisms for alcohol-induced liver injury may not be the same for non-alcohol related liver injuries [50]. Also, the identified relationships may change among people with diagnosed liver diseases, such as liver cancer. In our study population, however, these people only accounted for 3%, and removing them did not materially change the associations. Moreover, taking steatosis-inducing medication may also influence liver metabolism, but this was not assessed in this study. Additionally, we measured associations only among those who reported having ingested caffeine. Finally, we conducted a stratification analysis by HSI, a tool originally developed to screen for non-alcoholic fatty liver disease (NAFLD) in Koreans. HSI has been shown to be associated with the stage of non-alcoholic fatty liver disease (NAFLD), with an area under the receiver-operating curve (AUROC) of 0.812 in a representative Korean population [31]. While the underlying principles, such as a higher score indicating elevated liver damage and an increased risk of NAFLD, are likely applicable to the broader U.S. population, further validation studies are necessary to confirm this assumption.

## 5. Conclusions

Serum bicarbonate, serum phosphorus, and eGFR are inexpensive, accessible, and non-invasive metrics used in clinics. Urine caffeine metabolite indices are non-invasive and easier to apply compared to liver biopsy. To our knowledge, this is the first study to measure associations between these metrics and CYP1A2 activity. Our study contributes toward growing research on non-invasive ways to study how the liver helps regulate toward acid-base balance and with reduced renal function even among people without significant liver damage. Further studies using longitudinal data are necessary to understand the mechanisms behind the associations of these serum biomarkers and eGFR with liver metabolism. Novel, non-invasive, and sensitive biomarkers to measure early stage of acid-base balance are also needed.

## Data Availability

Data presented in this study is openly available online at [https://wwwn.cdc.gov/nchs/nhanes/continuousnhanes/default.aspx?BeginYear=2019 (accessed on 27 July 2023)].

## References

[1] Ando Y., Jou J.H. (2021). Nonalcoholic Fatty Liver Disease and Recent Guideline Updates. Clin. Liver Dis..

[2] Eslam M., Newsome P.N., Sarin S.K., Anstee Q.M., Targher G., Romero-Gomez M., Zelber-Sagi S., Wong V.W.-S., Dufour J.-F., Schattenberg J.M. (2020). A New Definition for Metabolic Dysfunction-Associated Fatty Liver Disease: An International Expert Consensus Statement. J. Hepatol..

[3] Estes C., Anstee Q.M., Arias-Loste M.T., Bantel H., Bellentani S., Caballeria J., Colombo M., Craxi A., Crespo J., Day C.P. (2018). Modeling NAFLD Disease Burden in China, France, Germany, Italy, Japan, Spain, United Kingdom, and United States for the Period 2016–2030. J. Hepatol..

[4] Smith A., Baumgartner K., Bositis C. (2019). Cirrhosis: Diagnosis and Management. Am. Fam. Physician.

[5] Piazzolla V.A., Mangia A. (2020). Noninvasive Diagnosis of NAFLD and NASH. Cells.

[6] Baumgartner K., Cooper J., Smith A., St Louis J. (2021). Liver Disease: Cirrhosis. FP Essent..

[7] Kisseleva T., Brenner D. (2021). Molecular and Cellular Mechanisms of Liver Fibrosis and Its Regression. Nat. Rev. Gastroenterol. Hepatol..

[8] Kalra R., Patel N., Arora P., Arora G. (2019). Cardiovascular Health and Disease Among Asian-Americans (from the National Health and Nutrition Examination Survey). Am. J. Cardiol..

[9] Ambühl P.M. (2011). Protein Intake in Renal and Hepatic Disease. Int. J. Vitam. Nutr. Res. Int. Z. Vitam. Ernahrungsforschung J. Int. Vitaminol. Nutr..

[10] Hakkola J., Hukkanen J., Turpeinen M., Pelkonen O. (2020). Inhibition and Induction of CYP Enzymes in Humans: An Update. Arch. Toxicol..

[11] Wijnen P.A.H.M., Op Den Buijsch R.A.M., Drent M., Kuipers P.M.J.C., Neef C., Bast A., Bekers O., Koek G.H. (2007). Review Article: The Prevalence and Clinical Relevance of Cytochrome P450 Polymorphisms. Aliment. Pharmacol. Ther..

[12] Guo J., Zhu X., Badawy S., Ihsan A., Liu Z., Xie C., Wang X. (2021). Metabolism and Mechanism of Human Cytochrome P450 Enzyme 1A2. Curr. Drug Metab..

[13] Gu L., Gonzalez F.J., Kalow W., Tang B.K. (1992). Biotransformation of Caffeine, Paraxanthine, Theobromine and Theophylline by cDNA-Expressed Human CYP1A2 and CYP2E1. Pharmacogenetics.

[14] Garduno A., Wu T. (2021). Tobacco Smoke and CYP1A2 Activity in a US Population with Normal Liver Enzyme Levels. Int. J. Environ. Res. Public. Health.

[15] Hopkins E., Sanvictores T., Sharma S. (2022). Physiology, Acid Base Balance. StatPearls.

[16] Katopodis P., Pappas E.M., Katopodis K.P. (2022). Acid-Base Abnormalities and Liver Dysfunction. Ann. Hepatol..

[17] Sacks G.S. (2004). The ABC’s of Acid-Base Balance. J. Pediatr. Pharmacol. Ther..

[18] Scheiner B., Lindner G., Reiberger T., Schneeweiss B., Trauner M., Zauner C., Funk G.-C. (2017). Acid-Base Disorders in Liver Disease. J. Hepatol..

[19] Seifter J.L., Chang H.-Y. (2016). Disorders of Acid-Base Balance: New Perspectives. Kidney Dis..

[20] NHANES NHANES 2009–2010: Lab Manual. https://wwwn.cdc.gov/nchs/data/nhanes/2009-2010/manuals/lab.pdf.

[21] NHANES NHANES 2009-2010: Physical Activity—Data Documentation, Codebook, and Frequencies. https://wwwn.cdc.gov/nchs/nhanes/2009-2010/PAQ_F.htm.

[22] NHANES NHANES 2009–2010: Smoking—Cigarette Use Data Documentation, Codebook, and Frequencies. https://wwwn.cdc.gov/nchs/nhanes/2009-2010/smq_f.htm.

[23] NHANES NHANES 2009–2010: Blood Pressure Data Documentation, Codebook, and Frequencies. https://wwwn.cdc.gov/nchs/nhanes/2009-2010/BPX_F.htm.

[24] NHANES NHANES 2009–2010: Body Measures Data Documentation, Codebook, and Frequencies. https://wwwn.cdc.gov/nchs/nhanes/2009-2010/BMX_F.htm.

[25] American Heart Association High Blood Pressure|American Heart Association. https://www.heart.org/en/health-topics/high-blood-pressure.

[26] NHANES NHANES 2009–2010: Dietary Interview—Individual Foods, First Day Data Documentation, Codebook, and Frequencies. https://wwwn.cdc.gov/Nchs/Nhanes/2009-2010/DR1IFF_F.htm.

[27] Nehlig A. (2018). Interindividual Differences in Caffeine Metabolism and Factors Driving Caffeine Consumption|Pharmacological Reviews. Pharmacol. Rev..

[28] NHANES NHANES 2009–2010: Caffeine & Caffeine Metabolites—Urine Data Documentation, Codebook, and Frequencies. https://wwwn.cdc.gov/Nchs/Nhanes/2009-2010/CAFE_F.htm.

[29] NHANES NHANES 2009–2010: Standard Biochemistry Profile Data Documentation, Codebook, and Frequencies. https://wwwn.cdc.gov/Nchs/Nhanes/2009-2010/BIOPRO_F.htm#Description_of_Laboratory_Methodology.

[30] Giglio D. (2014). A New Equation for Estimating Glomerular Filtration Rate in Cancer Patients. Chemotherapy.

[31] Lee J.-H., Kim D., Kim H.J., Lee C.-H., Yang J.I., Kim W., Kim Y.J., Yoon J.-H., Cho S.-H., Sung M.-W. (2010). Hepatic Steatosis Index: A Simple Screening Tool Reflecting Nonalcoholic Fatty Liver Disease. Dig. Liver Dis..

[32] Cohen R.D. (1991). Roles of the liver and kidney in acid—Base regulation and its disorders. Br. J. Anaesth..

[33] Almajid A.N., Sugumar K. (2023). Physiology, Bile. StatPearls.

[34] Hundt M., Basit H., John S. (2023). Physiology, Bile Secretion. StatPearls.

[35] Ishiguro H., Yamamoto A., Nakakuki M., Yi L., Ishiguro M., Yamaguchi M., Kondo S., Mochimaru Y. (2012). Physiology and Pathophysiology of Bicarbonate Secretion by Pancreatic Duct Epithelium. Nagoya J. Med. Sci..

[36] Rajkumar P., Pluznick J.L. (2018). Acid-Base Regulation in the Renal Proximal Tubules: Using Novel pH Sensors to Maintain Homeostasis. Am. J. Physiol. Ren. Physiol..

[37] Adeva-Andany M.M., Pérez-Felpete N., Fernández-Fernández C., Donapetry-García C., Pazos-García C. (2016). Liver Glucose Metabolism in Humans. Biosci. Rep..

[38] Barmore W., Azad F., Stone W.L. (2023). Physiology, Urea Cycle. StatPearls.

[39] Ogobuiro I., Tuma F. (2023). Physiology, Renal. StatPearls.

[40] Mao S.A., Glorioso J.M., Nyberg S.L. (2014). Liver Regeneration. Transl. Res. J. Lab. Clin. Med..

[41] Musso G., Gambino R., Tabibian J.H., Ekstedt M., Kechagias S., Hamaguchi M., Hultcrantz R., Hagström H., Yoon S.K., Charatcharoenwitthaya P. (2014). Association of Non-Alcoholic Fatty Liver Disease with Chronic Kidney Disease: A Systematic Review and Meta-Analysis. PLoS Med..

[42] Mantovani A., Zaza G., Byrne C.D., Lonardo A., Zoppini G., Bonora E., Targher G. (2018). Nonalcoholic Fatty Liver Disease Increases Risk of Incident Chronic Kidney Disease: A Systematic Review and Meta-Analysis. Metabolism..

[43] Umbro I., Baratta F., Angelico F., Del Ben M. (2021). Nonalcoholic Fatty Liver Disease and the Kidney: A Review. Biomedicines.

[44] Ryou M., Stylopoulos N., Baffy G. (2020). Nonalcoholic Fatty Liver Disease and Portal Hypertension. Explor. Med..

[45] Mazidi M., Mikhailidis D.P., Dehghan A., Jóźwiak J., Covic A., Sattar N., Banach M. (2021). The Association between Coffee and Caffeine Consumption and Renal Function: Insight from Individual-Level Data, Mendelian Randomization, and Meta-Analysis. Arch. Med. Sci. AMS.

[46] Stephens J.C., Schneider J.A., Tanguay D.A., Choi J., Acharya T., Stanley S.E., Jiang R., Messer C.J., Chew A., Han J.H. (2001). Haplotype Variation and Linkage Disequilibrium in 313 Human Genes. Science.

[47] Fekete F., Mangó K., Minus A., Tóth K., Monostory K. (2022). CYP1A2 mRNA Expression Rather than Genetic Variants Indicate Hepatic CYP1A2 Activity. Pharmaceutics.

[48] Nelson M.R., Marnellos G., Kammerer S., Hoyal C.R., Shi M.M., Cantor C.R., Braun A. (2004). Large-Scale Validation of Single Nucleotide Polymorphisms in Gene Regions. Genome Res..

[49] Kall M.A., Vang O., Clausen J. (1996). Effects of Dietary Broccoli on Human in Vivo Drug Metabolizing Enzymes: Evaluation of Caffeine, Oestrone and Chlorzoxazone Metabolism. Carcinogenesis.

[50] Toshikuni N., Tsutsumi M., Arisawa T. (2014). Clinical Differences between Alcoholic Liver Disease and Nonalcoholic Fatty Liver Disease. World J. Gastroenterol..

